# The Kinase STATE TRANSITION 8 Phosphorylates Light Harvesting Complex II and Contributes to Light Acclimation in *Arabidopsis thaliana*

**DOI:** 10.3389/fpls.2019.01156

**Published:** 2019-09-19

**Authors:** Paolo Longoni, Iga Samol, Michel Goldschmidt-Clermont

**Affiliations:** ^1^Department of Botany and Plant Biology, University of Geneva, Geneva, Switzerland; ^2^Institute of Biology, University of Neuchâtel, Neuchâtel, Switzerland; ^3^Institute of Biochemistry, University of Warsaw, Warsaw, Poland; ^4^Institute of Genetics and Genomics of Geneva (iGE3), University of Geneva, Geneva, Switzerland

**Keywords:** photosynthesis, protein phosphorylation/dephosphorylation, electron transport, thylakoids, light-harvesting complex II (LHCII), state transitions

## Abstract

Phosphorylation of the light-harvesting complex II (LHCII) is a central trigger for the reorganization of the photosynthetic complexes in the thylakoid membrane during short-term light acclimation. The major kinase involved in LHCII phosphorylation is STATE TRANSITION 7 (STN7), and its activity is mostly counteracted by a thylakoid-associated phosphatase, PROTEIN PHOSPHATASE 1/THYLAKOID ASSOCIATED PHOSPHATASE 38 (PPH1/TAP38). This kinase/phosphatase pair responds to the redox status of the photosynthetic electron transport chain. In *Arabidopsis thaliana*, Lhcb1 and Lhcb2 subunits of the LHCII trimers are the major targets of phosphorylation and have different roles in the acclimation of the photosynthetic machinery. Another antagonistic kinase and phosphatase pair, STATE TRANSITION 8 (STN8) and PHOTOSYSTEM II PHOSPHATASE (PBCP) target a different set of thylakoid proteins. Here, we analyzed double, triple, and quadruple knockout mutants of these kinases and phosphatases. In multiple mutants, lacking STN7, in combination with one or both phosphatases, but not STN8, the phosphorylation of LHCII was partially restored. The recovered phosphorylation favors Lhcb1 over Lhcb2 and results in a better adaptation of the photosynthetic apparatus and increased plant growth under fluctuating light. This set of mutants allowed to unveil a contribution of STN8-dependent phosphorylation in the acclimation to rapid light variations.

## Introduction

The conversion of photon energy into chemical energy drives the supply of organic compounds to the biosphere. It occurs in the thylakoid membrane through the action of a set of protein/pigment complexes capable of oxidizing water to oxygen, producing reducing agents that are used in plant metabolism. Simultaneously, electron transport leads to generation of a proton gradient across the thylakoid membrane, which energizes the ATP-synthase complex for ATP synthesis. The thylakoid membrane harbors several protein-pigment complexes involved in photosynthetic electron transport. Following the linear electron flow from water to NADPH these include photosystem II (PSII), cytochrome *b6f* (cyt*b6f*) and photosystem I (PSI). The photosystems are composed of a complex of core proteins, and the associated light harvesting complexes (LHCs). LHC II serves PSII, while both LHCI and LHCII function as PSI peripheral antennae; the LHCs capture light and transfer the energy towards the reaction centers of the photosystem cores. The structure and organization of LHCII appear to have permitted a fundamental step in evolution that allowed photosynthetic organisms to conquer the land environment, overcoming its constraints such as elevated oxygenation, low humidity and high irradiance (Ballottari et al., 2012). The major LHCII subunits can associate as trimers, which is the most abundant form in which they are found in the thylakoid membrane. In contrast, the minor LHCII subunits are usually monomeric and associated with PS II, connecting the external trimers to the reaction center. The LHCII proteins ensure rapid energy transfer towards the reaction centers by funneling light energy and allowing the following reactions to occur efficiently. It has been shown that this antenna system permits the transfer of photon energy to the reaction center, and thus charge separation, in 140 picoseconds (Caffarri et al., 2011). The rapid energy transfer ensures the sustained excitation of PSII that is necessary to perform the two photochemical turnovers required for the reduction of Q_B_ (PQ, plastoquinone) to Q_B_^2−^, which is further protonated to plastoquinol (PQH_2_). The latter acts in thylakoid membrane as the electron carrier to cyt*b_6_f*, also contributing to the formation of the proton gradient across the thylakoid membrane (Barber, 2016). In excess light condition, the antenna system acts dissipating the energy excess by a process known as nonphotochemical quenching (NPQ); this protects the two photosystems reaction centers and requires both the monomeric and the trimeric LHCII (Dall’Osto et al., 2017).

In limiting light conditions (e.g. under a canopy), the antenna system, devoted to light capture, must maximize and balance the funneling of energy towards the reaction centers. Under these conditions, changes in light quality can alter the excitation equilibrium between PSI and PSII. If the long wavelength light (far-red light) is enriched, it will preferentially excite PSI which contains more chlorophyll *a* and stable red-shifted pigments (Wientjes et al., 2012). Conversely, blue light will favor PSII by preferentially exciting LHCII, which is enriched in chlorophyll *b*. Changes in the equilibrium between the activities of the two photosystems are detected by the kinase STATE TRANSITION 7 (STN7). This kinase, through its interaction with the cyt*b_6_f*, responds to alterations of the balance between the oxidized (PQ) and reduced (PQH_2_) forms of plastoquinone (Shapiguzov et al., 2016; Dumas et al., 2017). Furthermore, the phosphorylation status of the STN7 kinase may affect its own proteolytic turnover and activity (Willig et al., 2011; Trotta et al., 2016).The principal described targets of the STN7 kinase are LHCII subunits. This kinase can phosphorylate a threonine in the N-terminal part of the LHCII proteins, causing the detachment of the antenna complex from PSII and its connection to PSI. This reversible process is known as state transitions (Rochaix et al., 2012). The *stn7* mutant is completely unable to reorganize the antenna system between the two photosystems (Bellafiore et al., 2005). State transitions are reversible through the action of a specific counteracting phosphatase named PROTEIN PHOSPHATASE 1/THYLAKOID-ASSOCIATED PHOSPHATASE 38 (PPH1/TAP38). Although state II to state I transition is abolished in the mutants of this phosphatase, the de-phosphorylation of the antenna can still occur, but at a much lower rate compared to the wild-type control (Pribil et al., 2010; Shapiguzov et al., 2010). A second pair of kinase and phosphatase is important in the regulation of the thylakoid phospho-proteome. The kinase is a paralog of STN7 and has therefore been called STATE TRANSITION 8 (STN8). This kinase is primarily responsible for the phosphorylation of subunits of the PSII core (PsbA/D1, PsbC/CP43, PsbD/D2 and PsbH) (Bonardi et al., 2005; Vainonen et al., 2005; Reiland et al., 2011). The counteracting PHOTOSYSTEM II CORE PHOSPHATASE (PBCP) is specifically implicated in the de-phosphorylation of the main STN8 targets (Samol et al., 2012). However, plants overexpressing this phosphatase also show an effect on the level of LHCII phosphorylation and on the kinetics of state transitions (Samol et al., 2012). It is only in the *stn7/stn8* double-mutant background that the phosphorylation of both LHCII and PSII core subunits is almost completely suppressed (Tikkanen et al., 2010). This suggests that the two kinases have a partially overlapping target specificity, therefore, a possible further distinction of their contributions to photosynthetic acclimation may derive from their different localization in the thylakoid membranes, STN7 being more abundant in the stromal lamellae and STN8 in the grana core (Wunder et al., 2013).

In *Arabidopsis thaliana* the major LHCII trimers are composed of three isoforms: Lhcb1, Lhcb2 and Lhcb3. This allows different combinations of LHCII trimers to have different characteristics and association with the photosystems (Galka et al., 2012). The three isoforms of the major LHCII trimers have distinct roles in photosynthetic acclimation. Lhcb1 is the most abundant isoform, it can be phosphorylated on a N-terminal threonine residue (Thr 3), and a lack of this isoform reduces the inducible heat dissipation of excess light (NPQ) (Pietrzykowska et al., 2014). Lhcb2 can also be phosphorylated at a N-terminal threonine (Thr 3), the sequence surrounding the phosphorylation site is conserved across higher plants, and it has been shown that Lhcb2 phosphorylation is central for the association of LHCII trimers to PSI for state transitions (Leoni et al., 2013; Pietrzykowska et al., 2014; Crepin and Caffarri, 2015; Longoni et al., 2015; Pan et al., 2018) and is involved in the migration of photosynthetic complexes between grana stacks and stroma lamellae (Rantala and Tikkanen, 2018). Finally, Lhcb3 is not phosphorylated and lacks an obvious phosphorylation site. Mutation of Lhcb3 affects the kinetics of LHCII reorganization during state transitions, and the binding of an LHCII trimer to the monomeric antenna Lhcb6 in PSII-LHCII complexes (Damkjaer et al., 2009; Su et al., 2017).

Here, we show that a mutant lacking the STN7 kinase can partially recover the phosphorylation of the major LHCII isoforms upon further removal of the counteracting phosphatases PPH1/TAP38 and PBCP. This phosphorylation is dependent on the STN8 kinase and shows a different regulation compared to the wild type. The recovered phosphorylation correlates with an increase of the plant’s ability to adapt to light variations.

## Materials and Methods

### Plant Material and Light Conditions

*Arabidopsis thaliana* ecotype Columbia (hereafter Arabidopsis) seeds were germinated on Murashige-Skoog agar plates at 22°C under white light (70 µmol s^−1^ m^−2^) with 16h daylight in a growth chamber (Percival CU36L5). For the experiments in soil the Arabidopsis plantlets were transferred after 5 days to pots, and grown at 22°C and 65% RH under white light (70 µmol s^−1^ m^−2^) with 12h daylight. After 5 days of acclimation the plants were either maintained in constant light or exposed to fluctuating light (4 min 50 µmol s^−1^ m^−2^ and 1 min 500 µmol s^−1^ m^−2^) under custom LED panels (PHYTOTRONIC EVO FutureLED) (**Supplementary Figure 1**). The multiple mutant lines were obtained by crossing of the previously described SALK insertional lines *stn7* (Bellafiore et al., 2005), *pph1-3* (Shapiguzov et al., 2010), *pbcp-1* (Samol et al., 2012) and *stn8* (Vainonen et al., 2005). The insertions in the multiple lines were genotyped by PCR as previously described (Samol et al., 2012). The rosette surface was measured with ImageJ software, using a color threshold to distinguish the leaves from the background. The surface in pixels was converted to cm^2^ using a scale.

### Protein Analysis and Immunodetection

Sample preparation and protein separation by electrophoresis in polyacrylamide gels containing Phos-tag were performed as previously described (Longoni et al., 2015). For the detection with the phospho-specific antibodies the samples were separated by Tris-Gly SDS-PAGE in 12% acrylamide gels and then transferred to a nitrocellulose membrane. For the immunodetection the following antibodies from Agrisera were employed: Lhcb1 (AS09 522), Lhcb2 (AS01 003), Lhcb1-P (AS13 2704), Lhcb2-P (AS13 2705), PsbA (AS05 084) and PsbA-P (AS13 2669) and actin (AS13 2640). The detection of the phosphorylated proteins was performed by separation on a Tris-Gly acrylamide gel 12% supplemented with 6M Urea, the proteins were transferred on a nitrocellulose membrane and decorated with a Phospho-threonine antibody supplied by Cell Signaling (9381). After incubation with the secondary antibody (Promega) and ECL reagent, the chemoluminescence was detected using a LAS-4000 Mini cooled CCD camera. Band intensity was measured with ImageJ software. Separation of supercomplexes by blue native polyacrylamide gel electrophoresis was performed as previously described (Longoni et al., 2015). Thylakoid preparation was performed according to (Arnold et al., 2014); the grana stacks and the stroma lamellae fraction were obtained by solubilization of the thylakoid preparation (0.5 mg mL^−1^) with 1% digitonin (Serva), followed by sequential ultracentrifugation 40,000*g* for 40 min to pellet the grana and 180,000*g* for 90 min to pellet the stroma lamellae fraction.

### Chlorophyll Fluorescence Analysis

Room temperature chlorophyll fluorescence was monitored with a FluorCam 800MF kinetic imaging fluorometer (PhotoSystems Instruments). Each fluorescence experiment was performed after 15 min of dark adaptation. At the end of this dark phase, the minimal fluorescence in the dark was recorded (F_O_), while the maximal fluorescence was measured with a saturating light pulse (F_M_). For state transition assessment, the chlorophyll fluorescence was measured on plants exposed to 10 min actinic red light supplemented with far-red light (Light 1) followed by 10 min of pure red light (Light 2). At the end of each light interval F_M_1 (at the end of the first 10 min) and F_M_2 (at the end of the second light phase) were measured. After the second light step the lights were turned off, and far-red flashes were used to oxidize the ETC, after 2 min the minimal fluorescence after light (F_O_′) was measured. Fluorescence values (Fs) before F_M_1 and F_M_2 were taken as steady state fluorescence in light 1 (Fs_STI) and light 2 (Fs_STII). The fluorescence value recorded just after turning-off the far-red light was labeled as Fp_STI. Derived values were calculated as follows: 1-qP = 1-((F_M_2 - Fs_STII)/(F_M_2 - F_O_′)), qT(Fs) = (Fp_STI-Fs_STII)/Fs_STII. Similar values were recorded and calculated for the fluctuating light transect. At the end of each light phase, low light (Fs_LL1, Fs_LL2, Fs_LL3, Fs_LL4) or high light (Fs_HL1, Fs_HL2, Fs_HL3), the steady state fluorescence was recorded followed by a measurement of the maximal fluorescence in low light (F_M__LL1, F_M_ _LL2, F_M_ _LL3, F_M_ _LL4) and high light (F_M__HL1, F_M__HL2, F_M__HL3). The ΦPSII value was calculated for each light step, (i.e. ΦPSII for LL1 = ((F_M_LL1 - Fs_ LL1)/F_M__LL1)) this parameter was employed to avoid a dark period between the light phases which is necessary to measure F_O_′ and therefore calculate other derived parameters (Murchie and Lawson, 2013). The relative ETR was calculated from the ΦPSII after 1 min with increasing light intensities with the following formula: ETR = ΦPSII · 0.5 · 0.8 · PAR µmol s^−1^ m^−2^, assuming that the light absorption was equivalent between the genotypes and that there was no major difference in PSI/PSII stoichiometry.

## Results

### Absence of the Thylakoid Phosphatases Restores LHCII Phosphorylation in the *STN7* Mutant

**Figure 1:**
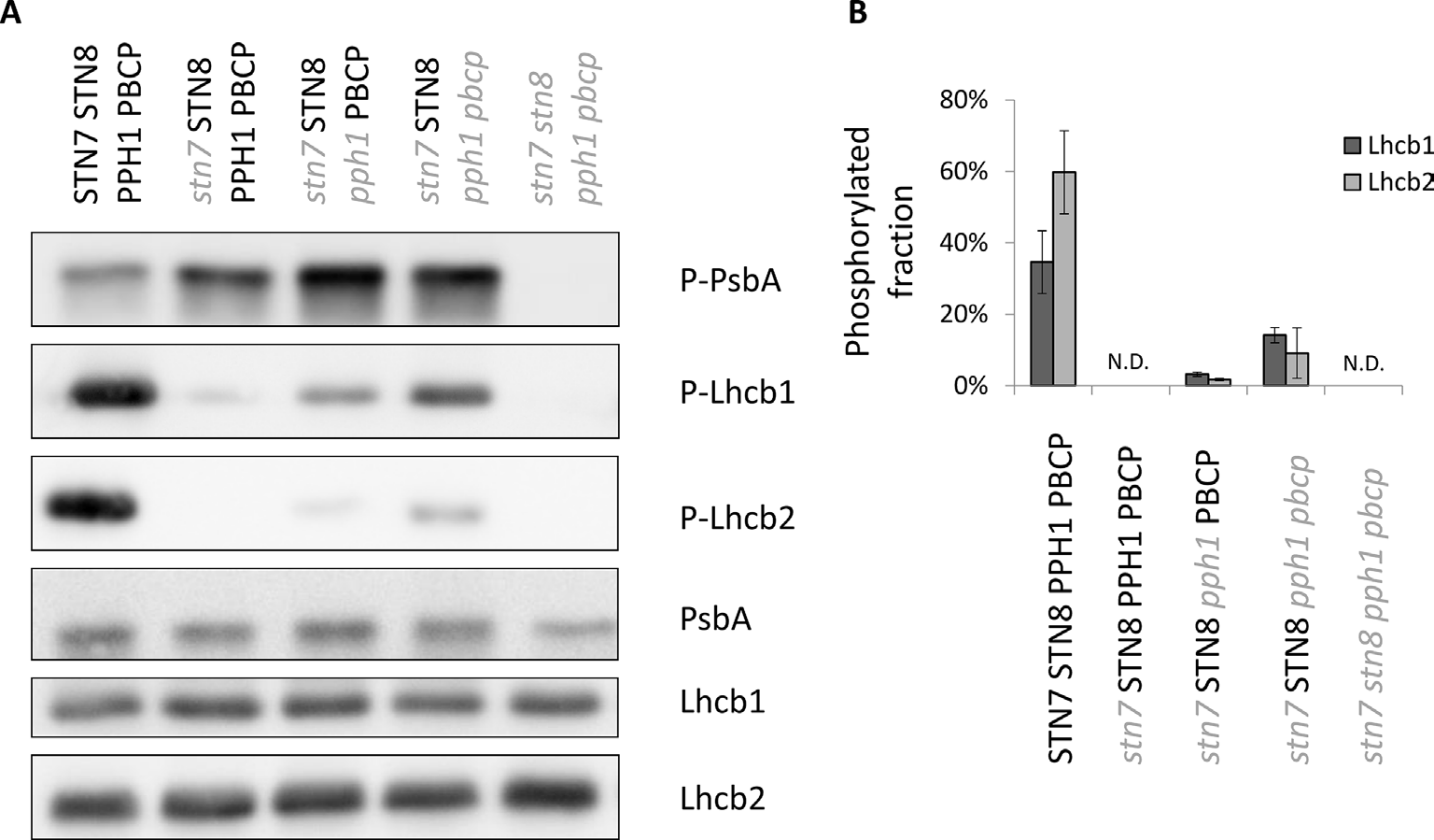
Differential recovery of the phosphorylation level of Lhcb1 and Lhcb2 in multiple mutants. **(A)** The level of phosphorylation of the LHCII antenna and the core protein PsbA/D1 of PSII was assessed by immunoblotting with antibodies specifically recognizing the phosphorylated peptides. As a control, the total accumulation of the protein was also assessed by immunoblotting. **(B)** The phosphorylation level of Lhcb1 and Lhcb2 in 15 day-old plantlets was measured by quantification of the immunoblot signal after separation by Phos-tag PAGE. The average level of phosphorylation under 100 µmol photons m^−2^ s^−1^ is reported with its standard deviation (n = 3). The phosphorylation levels in the mutants is different from the WT, p values of a Welch T-test of the difference between the double (*stn7/****STN8****/pph1/***PBCP**) and the triple (*stn7/****STN8****/pph1/pbcp*) mutants are shown.

The two major isoforms of the trimeric LHCII antenna are Lhcb1 and Lhcb2; both of them can be specifically phosphorylated at the N-terminus on a threonine residue. In wild-type plants grown under white light, both proteins are phosphorylated, although to a different extent (Longoni et al., 2015). Plants mutated for the kinase STN7 lost almost completely the phosphorylation of both isoforms (**Figure 1**). Upon removal of the counteracting phosphatase (TAP38/PPH1), the phosphorylation is partially restored. Interestingly, this recovery is uneven between the two isoforms: mainly Lhcb1 regains some phosphorylation in this double-mutant background (**Figure 1**). This effect becomes more pronounced when also the second phosphatase acting on thylakoid proteins (PBCP) is knocked out. Hereafter, the mutant acronyms will also include the wild-type alleles (in bold uppercase), to clearly show which kinases and phosphatases are present or not. Thus, for example, the triple mutant retaining only STN8 will be denoted (*stn7/****STN8****/pph1/pbcp*). In fact, the loss of both phosphatases in this triple mutant allows a higher level of phosphorylation of not only Lhcb1 but also Lhcb2. However, the phosphorylation level of Lhcb2 still remains much lower than in the wild-type control (**Figure 1**, **Supplementary Figure 2**). Furthermore, when the second major kinase acting on thylakoid proteins (STN8) is also knocked-out in the quadruple mutant (*stn7/stn8/pph1/pbcp*), the phosphorylation is completely lost (**Figure 1**), suggesting that this kinase is essentially responsible for the LHCII phosphorylation in the absence of STN7. The stacked mutations of the kinases and phosphatases involved in the regulation of thylakoid protein phosphorylation do not alter the accumulation of the target proteins, as the total level of Lhcb1, Lhcb2 and PsbA/D1 (a representative subunit of PSII) are not affected in these mutant lines (**Figure 1**). Furthermore, mutation of *STN7*, *PPH1* and *PBCP* do not influence the accumulation level of the STN8 kinase (**Supplementary Figure 3**). Since the two kinases, STN7 and STN8, do not have exactly the same localization in the thylakoid membrane (Wunder et al., 2013), we tested if the recovered LHCII phosphorylation repartition was affected. Upon thylakoid fractionation in grana stacks and stroma lamellae by digitonin, we detected the phosphorylated antenna present in both fractions. Consistently with previous reports (Rantala and Tikkanen, 2018), phosphorylated Lhcb1 was more abundant in the grana stacks, while phosphorylated Lhcb2 was enriched in the stroma lamellae in wild type as in the double (*stn7/****STN8****/pph1/****PBCP***) and the triple (*stn7/****STN8****/pph1/pbcp*) mutants (**Supplementary Figure 4**).

**Figure 2:**
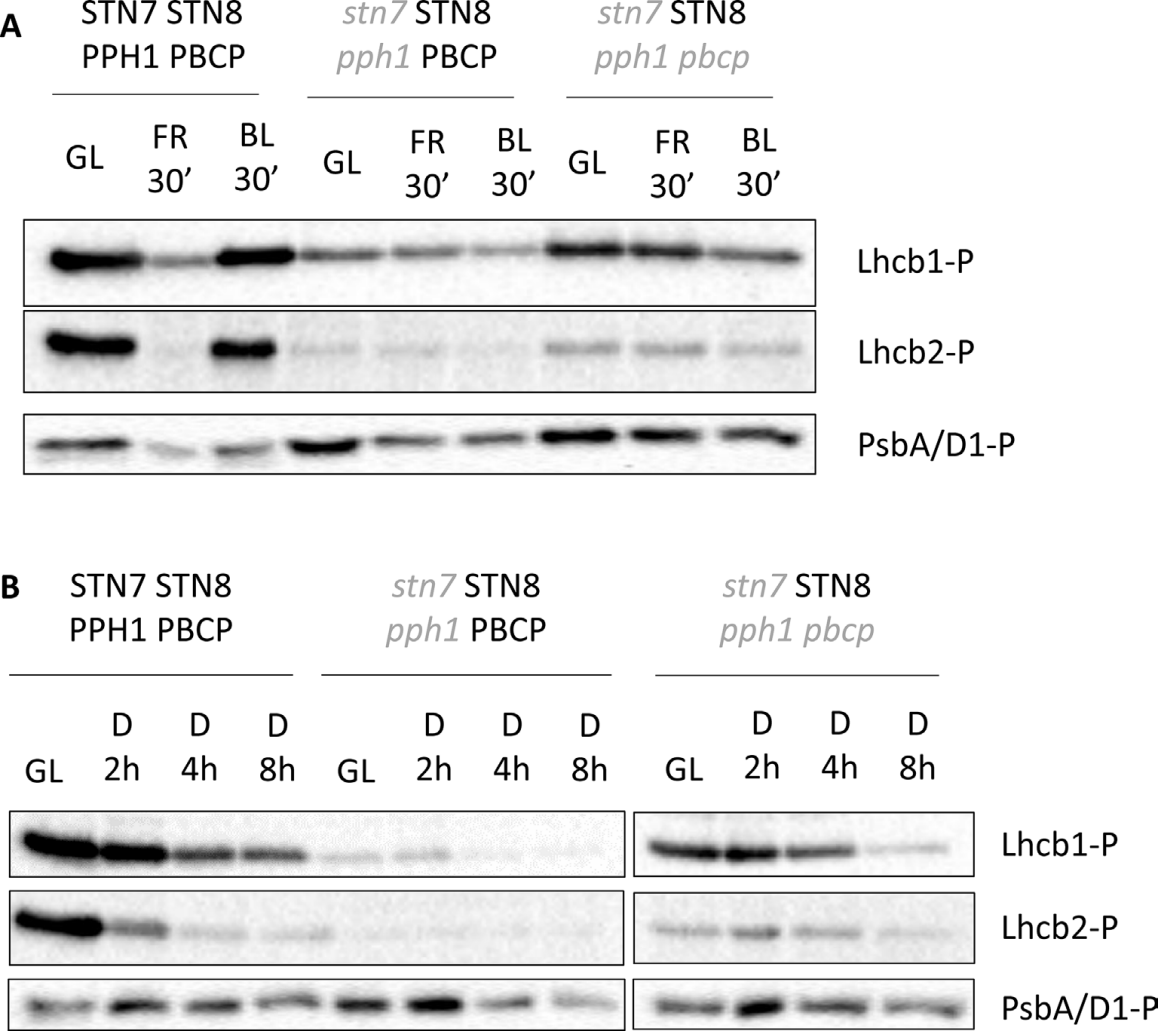
Lhcb1 and Lhcb2 phosphorylation dynamics after changes in light quality or prolonged darkness. **(A)** Total protein extracts from *Arabidopsis thaliana* mature leaves collected under white light (GL 100 µmol photons m^−2^ s^−1^), then exposed for 30 min to far red light (740 nm) and subsequently exposed to blue light (450 nm, 60 µmol photons m^−2^ s^−1^). **(B)** Total protein extracts from *Arabidopsis thaliana* mature leaves collected under white light (GL 100 µmol photons m^−2^ s^−1^) or after increasing time in total darkness (2, 4, and 8 h). The extracts were tested with antibodies recognizing the phosphorylated form of Lhcb1 (Lhcb1-P), Lhcb2 (Lhcb2-P) or PsbA/D1 (PsbA/D1-P).

An important feature of the phosphorylation of the LHCII antenna is its ability to respond quickly to changes in the redox status of the electron transport chain (ETC). When plants are exposed to a light condition that excites preferentially PSI (as occurs under 720 nm “far-red” light), the molecules involved in the ETC, and in particular plastoquinone, are oxidized, and this leads to the inactivation of STN7 followed by a de-phosphorylation of LHCII. The phosphorylation of the core proteins of PSII, which relies mostly on STN8 activity, is also strongly diminished under far-red light. The phosphorylation of the LHCII in our multiple mutants, which is dependent on STN8, was tested upon switching from white light to far-red light and then to blue light. The latter, exciting preferentially chlorophyll *b* in the antenna complex connected to PSII, triggered a re-phosphorylation of LHCII in wild-type plants (Longoni et al., 2015). The changes in the redox status of the ETC induced by blue light versus far-red light had only minor effects on the level of LHCII phosphorylation either in the double mutant (*stn7/****STN8****/pph1/****PBCP***) or in the triple mutant (*stn7/****STN8****/pph1/pbcp*) (**Figure 2A**, **Supplementary Figure 5A**), and if anything, phosphorylation in blue light was slightly lower. Also, exposing plants to high light intensity, shifting them from 50 to 500 µmol s^−1^ m^−2^ for 3 h did not affect the LHCII phosphorylation level in multiple mutants (**Supplementary Figure 6**). In darkness the redox state of plastoquinone is influenced by the activity of terminal plastid oxidase (PTOX), cytochrome *b559* of PSII and possibly by type II NAD(P)H-quinone oxidoreductase (NDC1) of chloroplast lipid droplets (Bondarava et al., 2003; Eugeni Piller et al., 2011), leading to a relatively slow oxidation of the plastoquinone pool. In darkness both the double (*stn7/****STN8****/pph1/****PBCP***) and the triple (*stn7/****STN8****/pph1/pbcp*) mutants had a slow rate of LHCII de-phosphorylation, possibly due to a residual counteracting activity of STN8 (**Figure 2B**, **Supplementary Figure 5B**).

### The Recovered LHCII Phosphorylation Does Not Allow State Transitions

**Figure 3:**
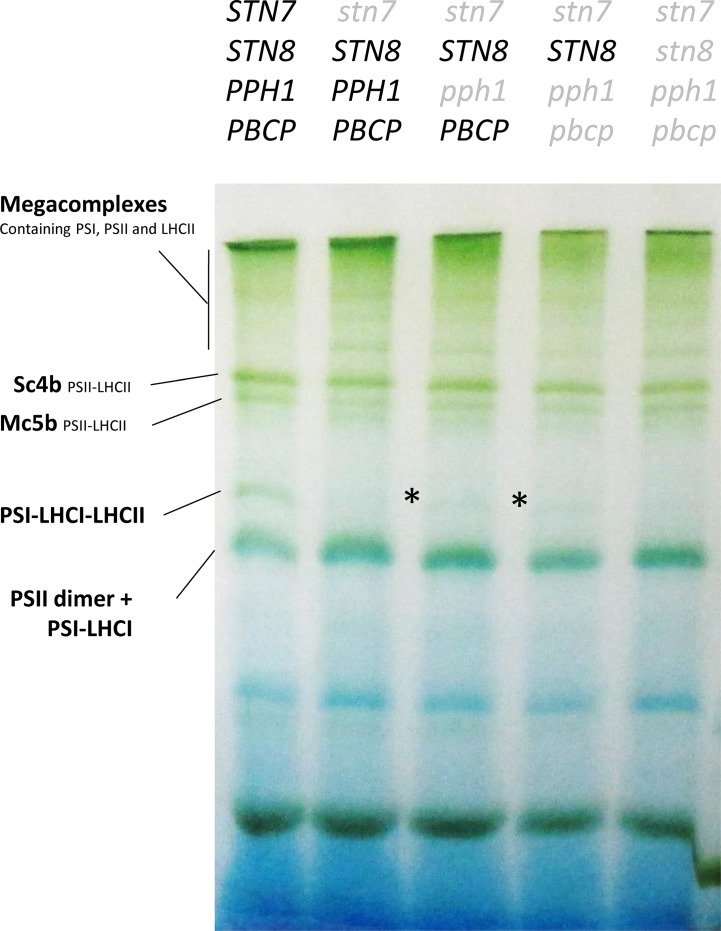
Analysis of supercomplexes from the stroma lamellae of the mutant lines. Supercomplexes were isolated with 1% digitonin from a thylakoid preparation of plants grown under constant light and analyzed by BN-PAGE. This digitonin fraction corresponds to supercomplexes present mostly in the stroma lamellae. The lack of antenna phosphorylation in the *stn7* mutant leads to the stabilization of supercomplexes migrating above the PSII-LHCII supercomplex, which are normally not clearly visible in the Col-0 background. These supercomplexes are present also in all the multiple mutants. A partial recovery of the PSI-LHCI-LHCII supercomplex (highlighted with an asterisk) can be observed in both the *stn7/****STN8****/pph1/***PBCP** mutant and the *stn7/****STN8****/pph1/pbcp* mutant. This supercomplex is completely absent in the quadruple mutant further lacking the STN8 kinase.

**Figure 4:**
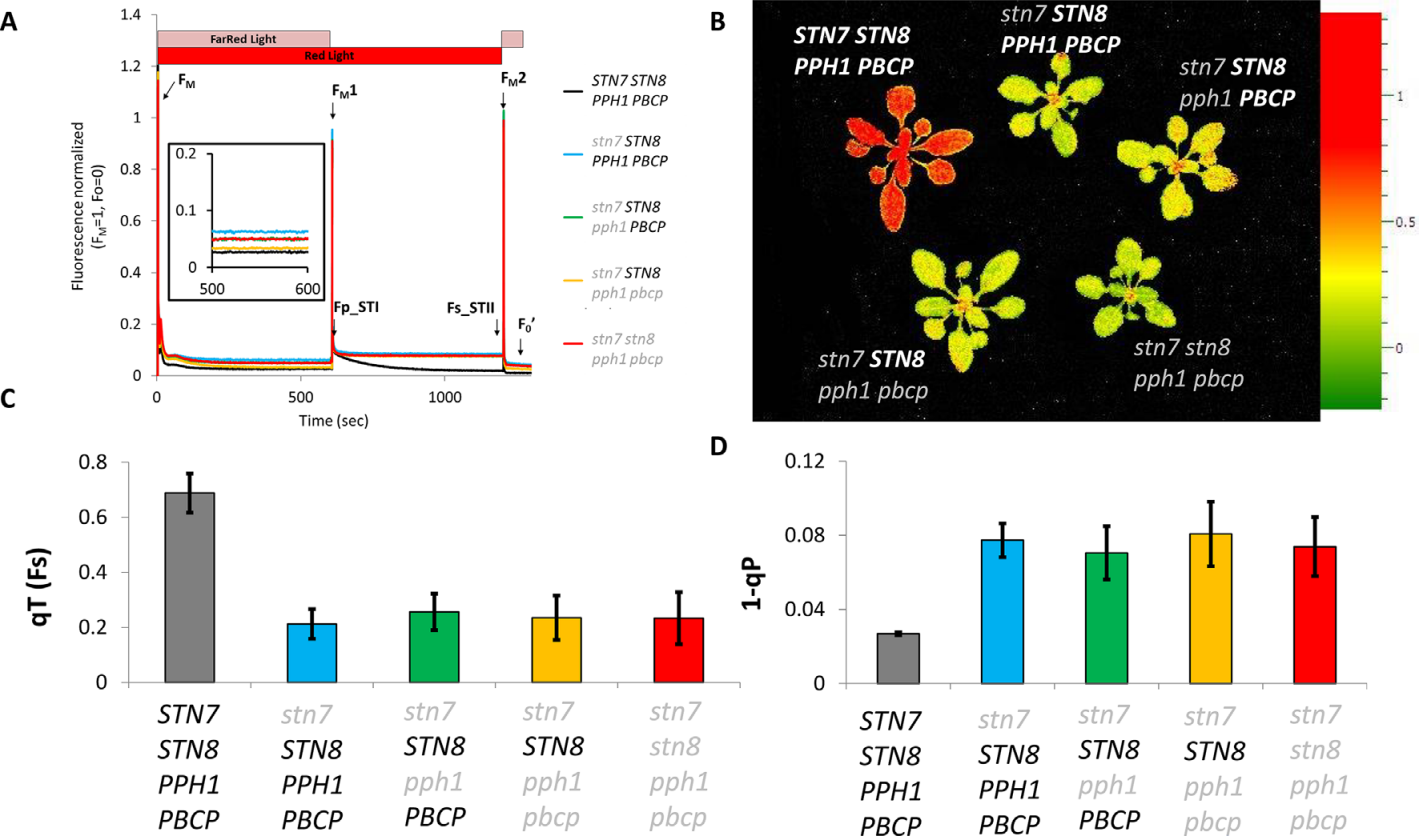
State transitions in *stn7* and multiple mutants**. (A)** The kinetics of state transitions were analyzed by continuously monitoring chlorophyll fluorescence with a FluorCam (MF800 PSI - PhotoSystems Instruments). After a preincubation of 30 min in darkness, actinic red light (represented as a red bar) and far-red light (represented as a pink bar) were turned on for 10 min to induce state 1 before maximal fluorescence (F_M_1) was measured with a 300ms saturating flash. Far-red light was then turned off for 10 min to induce a transition from state 1 to state 2, and maximal fluorescence (F_M_2) was again measured with a saturating flash. The curves (normalized to average F_M_ = 1, F_0_ = 0) are the average of three replicates of the protocol on a set of adult plants (4 weeks) of each genotype. F_0_’ is the minimal fluorescence measured in the dark after the light treatment. F_0_ was measured shortly before F_M_ and is not visible in the graph. **(B)** Heat map of the state transition measured in terms of variation of the fluorescence at the beginning (Fp_STI) and the end (Fs_STII) of the state 1 to state 2 transition. The two timepoints are marked in panel A with arrows, the scale represents the calculated value of qT(Fs) for every measured pixel which corresponds to (Fp_STI-Fs_STII)/Fs_STII. **(C)** Average value and standard deviation of the qT(Fs) parameter for the analyzed genotypes. **(D)** Average value and standard deviation of the 1-qP parameter representing the excitation pressure on PSII measured at the end of the red light phase (State II).

The phosphorylation of the Lhcb1 subunit may allow the release of excitation pressure on PSII upon changes in light quality, as was previously reported for lines lacking Lhcb2 (Pietrzykowska et al., 2014). Moreover, in the mutant lines presented above, Lhcb1 and Lhcb2 are present (**Figure 1**) and should form heterotrimers. Some of these should contain a phosphorylated Lhcb2 and thus potentially be capable of associating to PSI (Pan et al., 2018). The supercomplexes extracted from stromal lamellae of thylakoid membranes with digitonin were resolved by blue native PAGE. The supercomplexes formed in the double, triple, and quadruple mutant lines had a distribution that resembled closely the pattern observed in the single *stn7* mutant (**Figure 3**). Noteworthy, a faint band, ascribable to the PSI-LHCI-LHCII supercomplex, was visible in both the double and triple mutants. Both mutants had a very low level of Lhcb2 phosphorylation compared to the wild type (**Figure 1**) and, consistently, both mutants had a low amount of this specific supercomplex. Since a band corresponding in size to the PSI-LHCI-LHCII supercomplex was detectable in these mutant lines, the plants were tested for their ability to perform state transitions under changing light conditions. To this end, chlorophyll fluorescence from PSII was recorded at room temperature in plants that were exposed to far-red light superimposed on red light (State I light) and subsequently to red light only (State II light). This change in light spectrum creates an imbalance between PSI and PSII that is partially compensated by a transition to State II with the allocation of part of the LHCII antenna to PSI. We observed that, while the wild type shows a state transition, visible as a decrease in fluorescence from PSII during the red-light phase, all the mutants were incapable of responding to these light changes, and the fluorescence emission level remained constant during the red-light phase (**Figure 4**). It is also worth noting that, despite the recovery of Lhcb1 phosphorylation, all the mutant lines had the same level of excitation pressure on PS II at the end of the red-light phase, as denoted by the similar levels of 1-qP (**Figure 4D**). The over excitation of the PSII reaction center relative to the capacity of the ETC to accept electrons leads to an increased proportion of closed PSII centers even under this weak-light conditions, as was previously described for the single (*stn7/****STN8/PPH1/PBCP***) mutant (**Figure 4**) (Bellafiore et al., 2005). On the other hand, the steady state fluorescence level, normalized over F_M_, at the end of the far-red light phase (State I) was more variable, with only the (*stn7/****STN8/PPH1/PBCP***) mutant clearly higher than the wild type, while for the traces of the other mutant lines are closer to the wild-type value. This suggests that the recovered, and stable, LHCII phosphorylation in the multiple mutants may have a positive effect on diverting excitation to PSI, thus partially releasing the pressure on PSII at least under State I light conditions.

### Phosphorylation of Lhcb1 by STN8 Under Fluctuating Light

**Figure 5:**
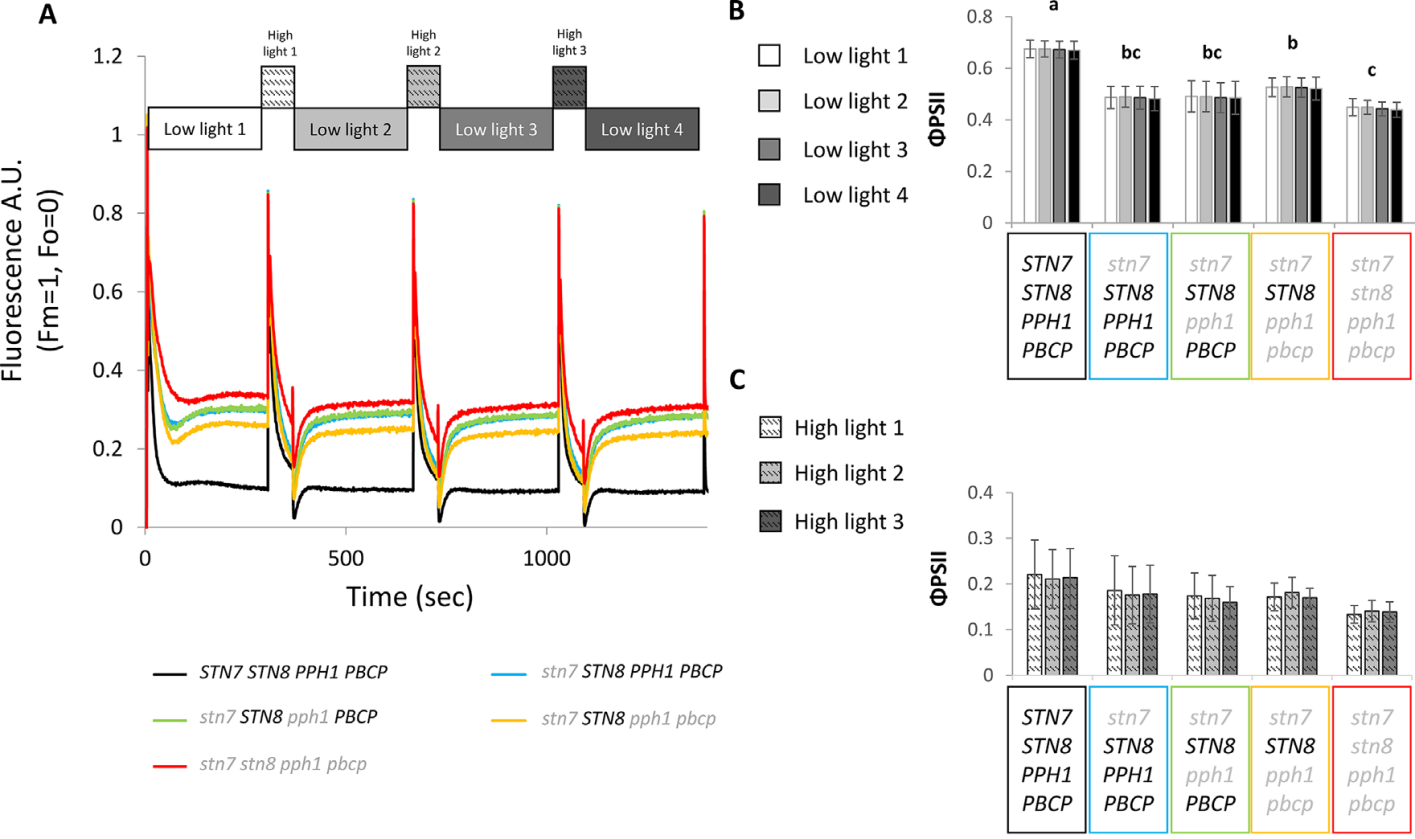
Room temperature fluorescence analysis under a fluctuating light regime. Plants grown under continuous white light (100 µmol photons m^−2^ s^−1^), were exposed to a variable light intensity regime of blue light (450 nm) composed of 4 min of low light (50 µmol photons m^−2^ s^−1^) and 1 min of high light (500 µmol photons m^−2^ s^−1^) in a FluorCam (MF800 PSI - PhotoSystems Instruments). **(A)** The graph shows the average of the room temperature fluorescence measured during this period for the different lines, the values have been normalized on F_M_ = 1 and on F_O_ = 0 (n = 5). **(B)** Values of the operating efficiency of photosystem II photochemistry (ΦPSII) calculated for the Col0 and mutant lines at the end of each low light phase Statistically different groups are marked with different letters (Student *t* test *P* < 0.05) error bars represent the standard deviation (n = 5). **(C)** Values of ΦPSII calculated at the end of each high-light phase. In both graphs the error bars represent the standard deviation (n = 5).

**Figure 6:**
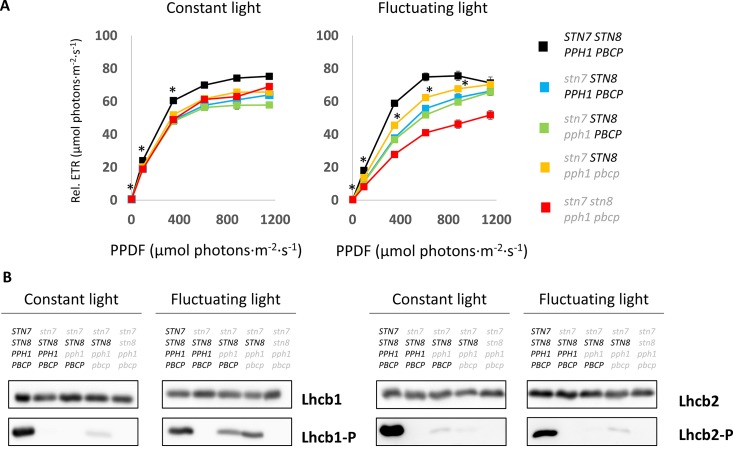
Photosynthetic efficiency and LHCII phosphorylation status upon growth under fluctuating light. **(A)** Electron transport efficiency Φ_PSII_ calculated from the room temperature chlorophyll fluorescence at increasing light intensities. The test was performed in parallel on 24-day old plants grown for 14 days under constant light (100 µmol photons m^−2^ s^−1^) (left) and fluctuating light (4 min at 50 µmol photons m^−2^ s^−1^ and 1 min at 500 µmol photons m^−2^ s^−1^) (right), plants were measured individually and the average ETR is reported (error bars indicate the S.E.). Light intensities at which the triple mutant (*stn7/****STN8****/pph1/pbcp*) relative ETR is statistically different from the other mutant lines and the wild type (Student *t* test *P* < 0.05) are highlighted by an asterisk. PPFD is the photoactive photon flux density measured as µmol of photons m^−2^ s^−1^. **(B)** The phosphorylation status of the two major LHCII isoforms assessed by immunodetection. The membrane blots of total protein extracts from plants grown under continuous or fluctuating light conditions were probed with antibodies against Lhcb1, Lhcb2 or their phosphorylated N-terminal peptides (Lhcb1-P or Lhcb2-P).

**Figure 7:**
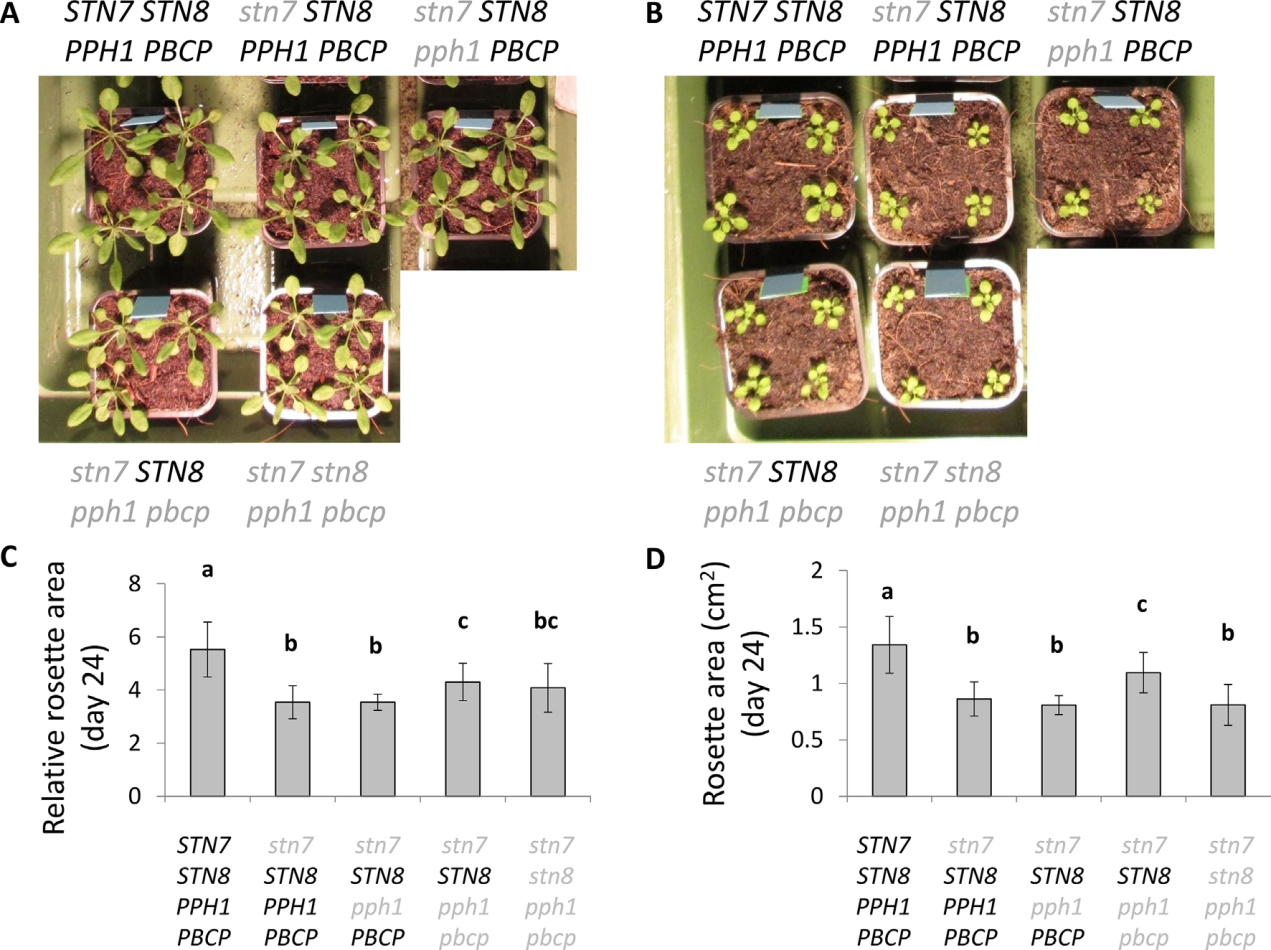
Growth of the plants and rosette area under fluctuating light intensity. **(A**, **B)**: Plants were grown under a long day cycle with during the day either **(A)** a constant light intensity (100 µmol photons m^−2^ s^−1^) or **(B)** a fluctuating light intensity with 4 min at 50 µmol photons m^−2^ s^−1^ and 1 min at 500 µmol photons m^−2^ s^−1^. The picture shows a representative set of plants for each tested genotype. **(C**, **D)**: Comparison of rosette size at day 24 for the different genotypes grown under the fluctuating light regime, expressed as relative rosette area compared to day 10.

The phosphorylation of LHCII has been reported to act as a protective mechanism against overreduction of the PSI reaction center (P700) under fluctuating light conditions (Grieco et al., 2012). It is proposed that the phosphorylated antenna transfers less energy to PSII and more to PSI and therefore avoids an overreduction of the electron transport chain. The double and triple mutants thus offered an opportunity to investigate the consequences of specifically Lhcb1 phosphorylation on ETC and on plant growth. The PSII quantum efficiency was examined by room-temperature fluorescence analysis under fluctuating light conditions (**Figure 5**). In these conditions, the operating efficiency of photosystem II photochemistry (ΦPSII) during the high-light phase showed no difference amongst the genotypes, while a slight increase in ΦPSII was observed in the plants expressing only STN8 (*stn7/****STN8****/pph1/pbcp*) (**Figure 5B**). However, this difference was significant only when compared with the mutant lacking both kinases and both phosphatases (*stn7/stn8/pph1/pbcp*). This suggests that the recovered, STN8-dependent phosphorylation has a small effect on the ETC efficiency. However, the recovered phosphorylation appeared to have a positive effect in preserving the ETC upon prolonged exposure to fluctuating light. In fact, we observed that, after ten days of exposure to this light regime, the ETR in the triple mutant mutant (*stn7/****STN8****/pph1/pbcp*) was significantly higher than in the single (*stn7/****STN8****/****PPH1****/****PBCP***) and in the double (*stn7/****STN8****/pph1/****PBCP***) mutants (**Figure 6A**). The difference was not detectable when plants were grown under a constant light regime. Interestingly, it appeared that the larger gain in efficiency occurred in the range of intensities comparable to those used in the fluctuating light conditions (50 to 500 µmol photons m^−2^ s^−1^), which is also a range of light intensity normally occurring in a natural environment (Schumann et al., 2017). The phosphorylation of Lhcb1 and Lhcb2 was assessed in all mutants after ten days growth under fluctuating light (**Figure 6B**). Under these light conditions, compared to constant light, the phosphorylation of Lhcb1 increased in the double mutant (*stn7/****STN8****/pph1/****PBCP***) and even more in the triple mutant (*stn7/****STN8****/pph1/pbcp*), while, in contrast, phosphorylation of Lhcb2 remained similar to the levels observed in constant light in all the mutant genotypes (**Figure 6B**). In contrast to Lhcb1 phosphorylation, no reproducible variation in PsbA/D1 phosphorylation level correlated with growth under fluctuating light in any of the mutant lines. To determine whether the recovered Lhcb1 phosphorylation had a physiological effect, plant growth was followed under prolonged fluctuating light conditions. As expected, all the mutant lines had a reduced rosette leaf area compared to wild type at the end of the period, consistent with their lower photosynthetic efficiency (**Figures 7A, B**). This defect was not visible in continuous light conditions, except for the quadruple mutant (**Supplementary Figure 7**). Interestingly, there was a small but significant increase of growth rate and final rosette leaf area for the mutant expressing only STN8 (*stn7/****STN8****/pph1/pbcp*), which has the highest Lhcb1 phosphorylation, compared to the other mutant lines (**Figures 7C, D**). Taken together, these results suggest that constitutive, STN8-dependent protein phosphorylation has a positive effect in maintaining photosynthetic efficiency and thus promoting plant growth.

## Discussion

### STN8 and PBCP Play a Role in the Control of LHCII Phosphorylation

Thylakoid proteins are highly phosphorylated and their phosphorylation status has a deep impact on the ability of the plants to acclimate to changing environments. The most striking effect on acclimation is observed in plants lacking the state transition kinase STN7. This mutant loses almost completely the phosphorylation of the major LHCII. This is in agreement with the widely accepted hypothesis that this kinase is responsible for their phosphorylation in wild-type plants, although an indirect effect cannot be ruled out (Bellafiore et al., 2005). It has been already reported, however, that a low level of LHCII phosphorylation is still detectable in the *stn7* mutant background, and that only by the further introduction of the *stn8* mutation, the phosphorylation of these proteins decreases below the detection level (Fristedt et al., 2010). The partial involvement of STN8 in LHCII phosphorylation is normally masked by the STN7 kinase and its counteracting phosphatase. Here, we could detect this activity of STN8 by knocking-out both PPH1/TAP38, which is the main phosphatase acting on LHCII, and PBCP, which has been reported to act mainly on the PSII core (Pribil et al., 2010; Shapiguzov et al., 2010; Samol et al., 2012). While both STN7 and PPH1/TAP38 have been reported to be active on both Lhcb1 and Lhcb2, (Fristedt and Vener, 2011; Wei et al., 2015), the triple mutant (*stn7/****STN8****/pph1/pbcp*) revealed that STN8 is capable of phosphorylating the LHCII proteins with a preference for the Lhcb1 isoform. This preference may also be explained by the difference in the localization of the two phosphorylated isoforms in the thylakoid membrane. In fact, phosphorylated Lhcb1 appears to be more accumulated in the grana stacks, where the STN8 kinase is more abundant, while phosphorylated Lhcb2 seems to accumulate more in the stroma lamellae fraction (Wunder et al., 2013; Rantala and Tikkanen, 2018) (**Supplementary Figure 4**). It is interesting to note that both phosphorylated Lhcb1 and Lhcb2 are present in both fractions also of the double (stn7/**STN8**/pph1/**PBCP**) and triple (stn7/**STN8**/pph1/pbcp) mutants, which shows that STN8 can act on substrates present in both fractions of the thylakoid membrane. The recognition of the phosphorylated forms of Lhcb1 and Lhcb2 by the phospho-specific antibodies implies that the phosphorylation occurs at the same residues as in the wild type (*i.e.* Thr 3 of the mature protein). Interestingly, it appears that also PBCP is active on LHCII since its removal, in the triple (stn7/**STN8**/pph1/pbcp) mutant compared to the double (stn7/**STN8**/pph1/**PBCP**) mutant, results in an increase of the phosphorylation of both Lhcb1 and Lhcb2 (Liu et al., 2019). The observed activity of PBCP on phosphorylated LHCII is in agreement with the previous report that overexpression of this phosphatase leads to a delay in state I to state II transitions (Samol et al., 2012).

### LHCII Phosphorylation in Multiple Mutants Is Not Regulated by the Redox Poise of the PQ Pool

Lhcb2 is the antenna isoform that is primarily involved, through its N-terminal phosphorylation, in the association of the LHCII trimer to PSI in the PSI-LHCI-LHCII supercomplex (Crepin and Caffarri, 2015; Longoni et al., 2015; Pan et al., 2018). Thus, it is not surprising that Lhcb2 phosphorylation has a greater impact on the acclimation of the photosynthetic electron transport chain and thus essentially masks the contribution of phosphorylated Lhcb1. Compared to the single mutant (*stn7/****STN8/PPH1/PBCP***), the double mutant (*stn7/****STN8****/pph1/****PBCP***) and triple mutant, (*stn7/****STN8****/pph1/pbcp*) exhibit increased phosphorylation levels of Lhcb1, and to a lesser extent of Lhcb2 (**Figure 1**). By comparing these genotypes, it was possible to address contributions of the phosphorylation of Lhcb1 and Lhcb2 to the acclimation of the photosynthetic apparatus. In contrast to phosphorylation by STN7, we observed that the recovered phosphorylation by STN8 does not respond to the rapid changes in the redox status of the plastoquinone pool that are induced by changes in light quality or intensity (**Figure 2**, **Supplementary Figure 6**). The steady phosphorylation of the LHCII may depend on the lack of the counteracting phosphatases, and mainly PPH1/TAP38 which is the major player in LHCII dephosphorylation (Pribil et al., 2010; Shapiguzov et al., 2010). However, the level of Lhcb1 phosphorylation does increase when plants are exposed to fluctuating light (**Figure 6B**) suggesting that STN8 does respond to changes in the electron transport chain induced by changing light conditions. These observations point towards unique regulatory features of STN8, which have not been fully understood yet. Lack of redox-responsive phosphorylation likely explains the lack of state transitions (qT) as measured by chlorophyll fluorescence (**Figure 4**). Nevertheless, BN-PAGE analysis revealed the presence of a small amount of PSI-LHCI-LHCII in double (*stn7/****STN8****/pph1/****PBCP***) and triple (*stn7/****STN8****/pph1/pbcp*) mutants, but not in *stn7* (*stn7/****STN8****/****PPH1****/****PBCP***) nor in the quadruple (*stn7/stn8/pph1/pbcp*) mutant. This could be explained by the presence of a low level of phosphorylated Lhcb2 in the double and triple mutants, independently of the redox state. The phospho-threonine near the N-terminus of Lhcb2 is implicated in the binding of the LHCII trimer to PSI (Crepin and Caffarri, 2015; Longoni et al., 2015; Pan et al., 2018). However, phosphorylated Lhcb1 is excluded from the trimer that binds to PSI (Crepin and Caffarri, 2015; Longoni et al., 2015). Instead, phosphorylated Lhcb1 may contribute to LHCII mobility, the unstacking of the grana and/or the removal of the peripheral antenna from of PSII supercomplexes (Fristedt et al., 2010; Pietrzykowska et al., 2014; Puthiyaveetil et al., 2017). However, the level of excitation pressure (1-qP) measured in the different multiple mutant lines, suggests that, under low light condition, the constitutive phosphorylation of Lhcb1 does not significantly reduce the antenna functionally connected to PSII (**Figure 4D**). Considering the low level of Lhcb2 phosphorylation and the limited accumulation of the stable PSI-LHCII supercomplex, we may hypothesize that the difference between the multiple mutant lines arises from Lhcb1 phosphorylation leading some LHCII trimers to associate to PSI through LHCI. Furthermore, it was recently shown that a second LHCII trimer may bind to PSI at an alternative site, involving the LHCI antenna, and that this interaction may be functional even though the supercomplex being formed is not stable enough to be identified by BN PAGE (Benson et al., 2015; Bos et al., 2017; Yadav et al., 2017; Bressan et al., 2018). These observations raise the intriguing question of whether phosphorylation of Lhcb1 could possibly regulate the labile association of an LHCII trimer to LHCI. However, since the recovered LHCII phosphorylation is stable, either for the lack of phosphatases or by a different regulation of STN8 compared to STN7, we may expect the repartition of the antenna not to change in the time scale of the canonical state transitions (i.e. 10 min). This will lead to plants with no visible change in the fluorescence traces as observed (**Figure 4A**).

### STN8-Dependent Phosphorylation Has a Protective Effect Under Fluctuating Light

The formation of the stable PSI-LHCI-LHCII supercomplex has been shown to correlate with improved electron transport and thus resistance to increasing light intensity in wheat (Marutani et al., 2017). It has also been suggested that stable phosphorylation of LHCII may help plants to cope with rapid fluctuations in light intensities (Grieco et al., 2012). The rationale is that short term variations in light do not allow changes in the level of phosphorylation of LHCII. Furthermore *stn7* mutant plants, lacking the major actor of LHCII phosphorylation, show impaired photosynthetic efficiency upon exposure to fluctuating light conditions (Bellafiore et al., 2005; Grieco et al., 2012). Under fluctuating light, we observed that Lhcb1 was significantly phosphorylated, but not Lhcb2, in the double (*stn7/****STN8****/pph1/****PBCP***) and triple (*stn7/****STN8****/pph1/pbcp*) mutants. This allowed us to investigate the specific contribution of phospho-Lhcb1 to this acclimation. Indeed, the triple mutant (*stn7/****STN8****/pph1/pbcp*), where Lhcb1 phosphorylation was highest compared to the other *stn7*-derived genotypes, also had a lower value of steady state fluorescence, already under constant light. This suggests that in this genotype the electron transport chain is more oxidized allowing a larger part of the PSII population to be oxidized, and thus a higher quantum efficiency (**Figure 5**), possibly decreasing the antenna functionally connected to PSII or favoring the oxidation of the ETC by exciting PSI. Nevertheless, it is also important to remember that the contribution of phospho-Lhcb2, which is highly phosphorylated only in the WT, seems to be the most effective, so that in the wild-type plants the fraction of closed (reduced) PSII centers is significantly smaller than in all the other genotypes. This difference can only be observed during the “low-light” phase, suggesting that at higher light intensities the phosphorylation status of the LHCII has a minor effect in regulating the electron transport efficiency. This is consistent with the widely accepted model of plant acclimation to light, where state transitions, mediated by phosphorylation of LHCII, are important to improve photosynthetic efficiency under low light intensities, while at higher light intensities other processes play a major role (e.g. thermal dissipation) (Rochaix, 2014; Khuong et al., 2019). Furthermore, Lhcb1 phosphorylation was also promoted under fluctuating light condition (**Figure 6**), and this increase correlated with an improved ETR compared to the other mutant lines. Even such a small gain in photosynthetic efficiency led to increased growth when the conditions triggering this difference were reiterated over a long time period (**Figure 7**), similar to what has been shown in the case of a tobacco line engineered to more rapidly relax NPQ upon shifting from high to low light (Kromdijk et al., 2016). In fact, the growth relative to day 10 of the quadruple mutant seemed also to be slightly higher than the single mutant *stn7* and the double (*stn7/****STN8****/pph1/****PBCP***). This apparent paradox is explained by the fact that at day 10 after sowing, the rosette area of this mutant was already smaller. This suggests that in the quadruple mutant, growth is affected already at early stages of development. As a consequence, despite its higher growth rate relative to day 10, at the end of the experiment the average rosette area of the quadruple mutant (*stn7/stn8/pph1/pbcp*) was still smaller than both the wild type and the triple mutant (*stn7/****STN8****/pph1/pbcp*) (**Figure 7D**). Therefore, this data supports the idea that the recovered STN8-dependent phosphorylation has a positive effect in preserving the ETC under fluctuating light, and thus increases the photosynthetic efficiency. Since STN8 has targets other than LHCII, there remains a possibility that the phenotypes that were observed are not due to the recovered phosphorylation of Lhcb1 itself (Reiland et al., 2011; Schönberg et al., 2017). A possible candidate is Lhcb4 which is phosphorylated by STN8 in rice (Betterle et al., 2017), however, no extra band at the expected size for Lhcb4 was observed in the anti phospho-threonine immunoblot, nor a shifted band when the protein was tested after migration in a Phos-tag^™^ containing gel (**Supplementary Figure 6B**). It has also to be noted that we failed to detect any correlation between the phosphorylation of the main target of STN8, namely the core of photosystem II (e.g. PsbA/D1) (Vainonen et al., 2005), and growth under fluctuating light. Furthermore, because the effective cross-section of LHCII is a primary determinant of excitation pressure on PSII, assigning the phenotypic effects to Lhcb1 phosphorylation appears the most likely hypothesis. In any case, phosphorylation of Lhcb1 can be taken as a proxy for the kinase activity of STN8 in the triple mutant (*stn7/****STN8****/pph1/pbcp*).

### Concluding Remarks

In conclusion, here we have shown that STN8 is capable of phosphorylating the threonines near the N-terminus of the Lhcb1 and Lhcb2 isoforms of trimeric LHCII, and that the level of this phosphorylation depends essentially on the absence of the counteracting phosphatases PPH1/TAP38 and PBCP. Although these are the same threonines that are the targets of STN7, their STN8-dependent phosphorylation does not respond to changes in the redox status of the photosynthetic electron transport chain, and does not allow state transitions. Under fluctuating light conditions, the phosphorylation of Lhcb1 correlates with (i) a small but significant relaxation of the excitation pressure on PSII, and (ii) a better acclimation of the mutant plants to fluctuating light and a better preservation of photosynthetic electron transfer. This suggests that STN8-dependent phosphorylation, most likely phosphorylation of Lhcb1, has a positive effect on photosynthetic acclimation to changing light environments.

## Data Availability

The datasets generated for this study are available on request to the corresponding author.

## Author Contributions

PL and IS conducted the experiments. PL, IS, and MG-C designed the experiments and wrote the paper.

## Funding

This research was supported by the University of Geneva and the Swiss National Science Foundation (SNF 31003A_146300 and 31003A_179417).

## Conflict of Interest Statement

The authors declare that the research was conducted in the absence of any commercial or financial relationships that could be construed as a potential conflict of interest.
