# Supplementary material for: The Kinase STATE TRANSITION 8 Phosphorylates Light Harvesting Complex II and Contributes to Light Acclimation in Arabidopsis thaliana
Source: Front Plant Sci. 2019 Sep 19;10:1156. doi: 10.3389/fpls.2019.01156 (PMC6761601; doi:10.3389/fpls.2019.01156)
Supplement: Supplementary file 1 [file DataSheet_1.pdf]

Supplementary Material

Supplementary Figures

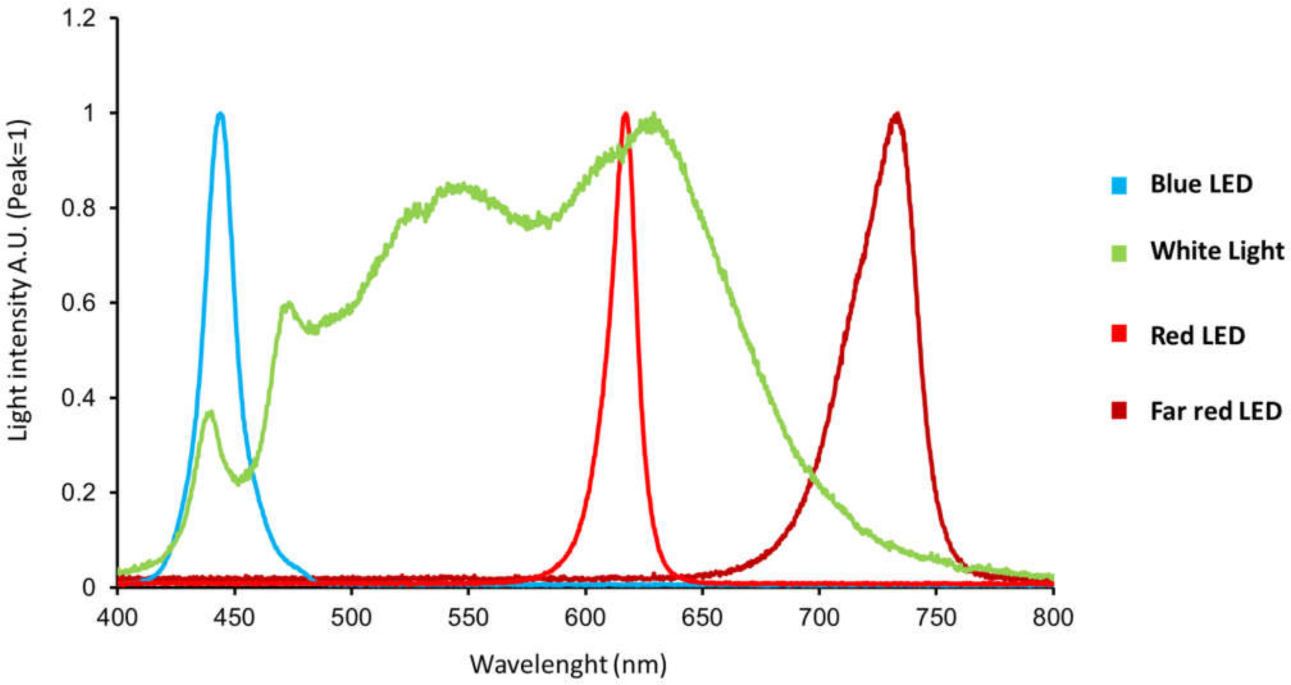

Supplementary Figure 1. Spectrum of light sources used for the experiments. Light emission spectrum between 400 and 800 nm for the light sources used for the experiments. Blue LED source was used to induce protein phosphorylation, Far red LED to induce de-phosphorylation and state 2 to state 1 transition during fluorescence measurement. Red LED were used for the measurement of fluorescence. White light is the spectrum of the light source used during growth under fluctuating light (PHYTOTRONIC EVO FutureLED); light fluctuation changed the intensity but not the spectrum. The spectra are normalized on maximum intensity (max=1), to allow a comparison of the different lights.

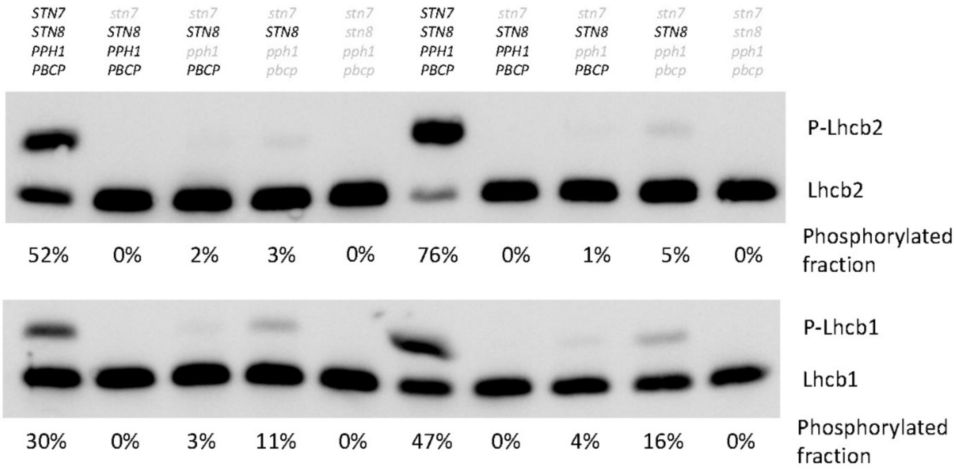

Supplementary Figure 2. Phos-tag™ PAGE separation and immunodetection of the two major LHCII isoforms Lhcb1 and Lhcb2.

Total protein extracts from 15-day old plantlets were prepared and separated on a double layer-gel containing Phos-tag™. The extracts were prepared from plants exposed to constant white light (100 µmol photons m^{-2} sec^{-1}). For each sample the upper band corresponds to the phosphorylated form of the protein, and the lower band to the non-phosphorylated one. The quantification of the signal was performed with ImageJ and the fraction of phosphorylated protein over the total is reported below each lane.

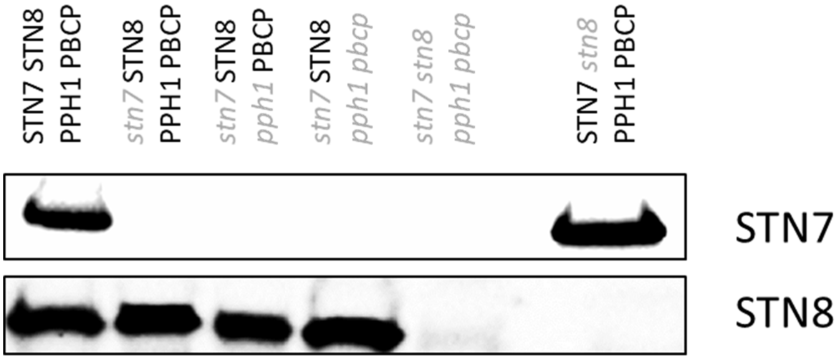

Supplementary Figure 3. Accumulation of STN7 and STN8 kinases in multiple mutants.

Total protein extracts from fully expanded mature leaves were prepared and separated on a SDS-PAGE. The blotted membranes were decorated with antibodies against the two kinases STN7 and STN8 and revealed by ECL.

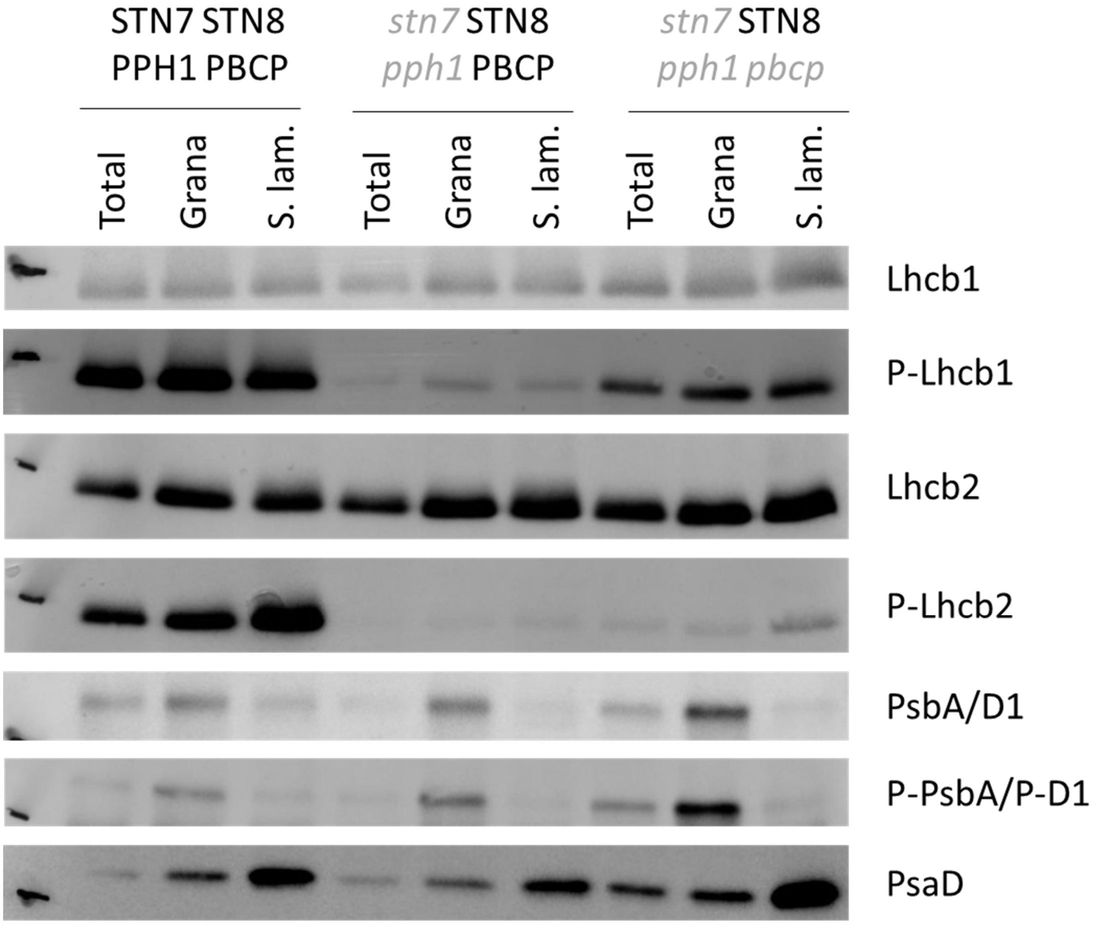

Supplementary Figure 4. Thylakoid fractionation and localization of the phosphorylated proteins.

Thylakoids were prepared from adult plants exposed to growth light (100 µmol photons m^{-2} sec^{-1}). The grana and stroma lamellae fractions were separated by digitonin solubilization (1%) and ultracentrifugation. Total proteins from each fraction were denatured with 2% SDS and separated by SDS-PAGE. Upon transfer to a nitrocellulose membrane the proteins were detected by mean of specific antibodies to reveal the presence of the two major LHCII isoforms (Lhcb1, Lhcb2) their phosphorylated form (P-Lhcb1, P-Lhcb2), the core subunit of PSII PsbA/D1 and its phosphorylated form. The PsaD subunit from PSI was additionally detected as a control of the fractions. Total thylakoids and the two fraction were loaded on equal chlorophyll basis (0.1 ug of total chlorophyll per well).

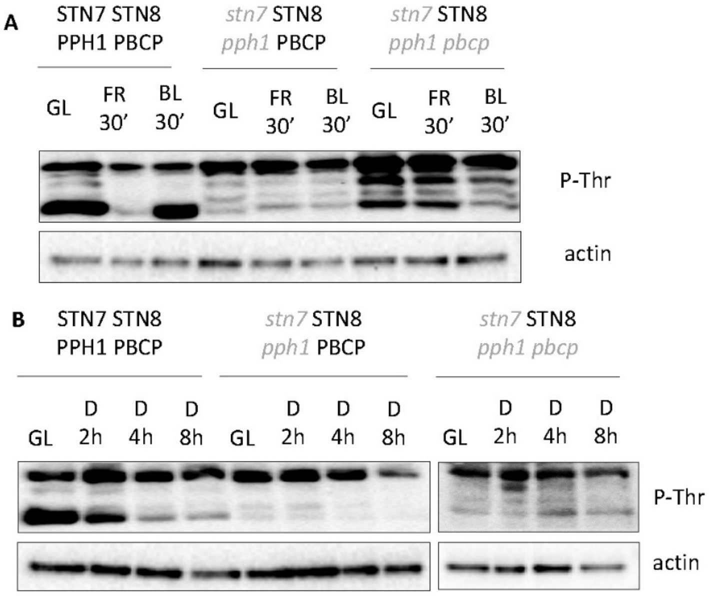

Supplementary Figure 5. Protein phosphorylation after changes in light quality or prolonged darkness.

Total protein samples were collected from mature leaves and tested with antibodies against phosphorylated threonine (P-Thr) and Actin as a loading control (actin). The samples are the same presented in Figure 2. (**A**) Protein extracts from plants grown under white light (GL 100 µmol photons m^{-2} sec^{-1}), then exposed for 30 minutes to far red light (740 nm) and subsequently exposed to blue light (450 nm, 60 µmol photons m^{-2} sec^{-1})) exposed to pure far-red light for 30’ followed by pure blue light for 30’ (**B**) Protein extracts from plants exposed to prolonged darkness.

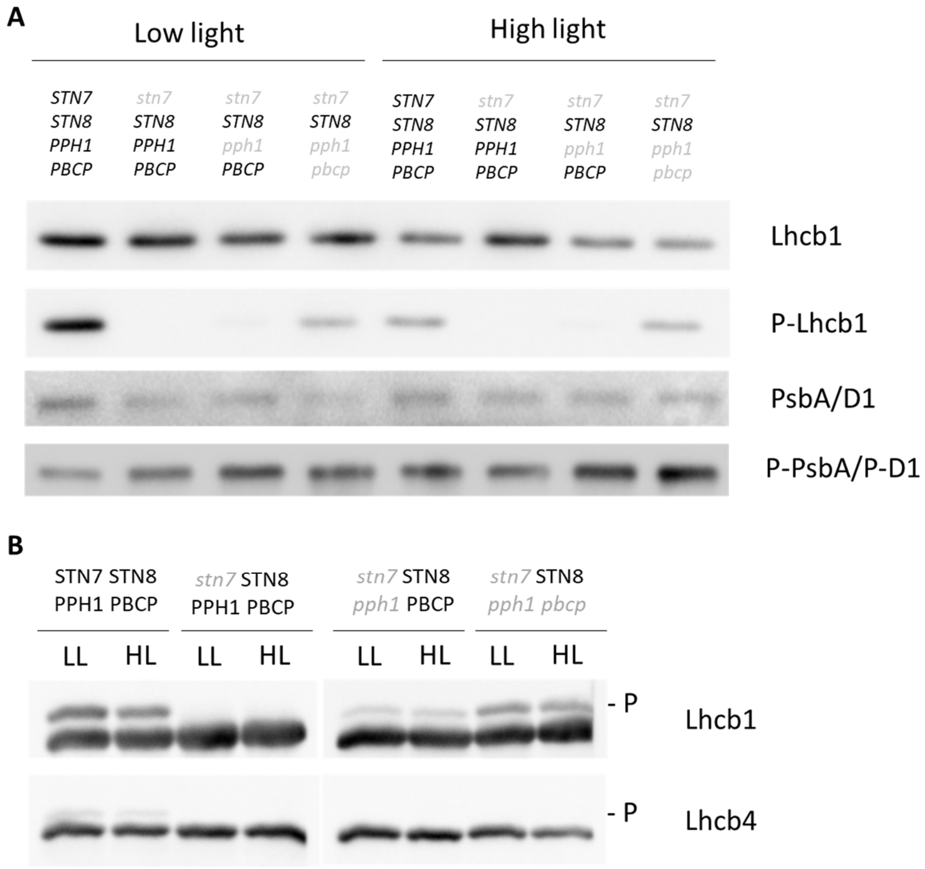

Supplementary Figure 6. LHCII and PSII core phosphorylation upon switching to high light.

Total protein extracts from fully expanded mature leaves collected after 3h of low light (50 µmol sec^{-1} m^{-2}) or collected after a following exposure of 3h under high light (500 µmol sec^{-1} m^{-2}) were prepared and separated on (**A**) SDS-PAGE for the detection of Lhcb1, PsbA/D1 and their respective phosphorylated form via specific antibody recognizing the two forms. (**B**) on a SDS-PAGE containing Phos-tag™ in order to separate the phosphorylated form by migration delay, the band corresponding to the phosphorylated form of the protein is indicated with a –P. LL: low light; HL: high light.

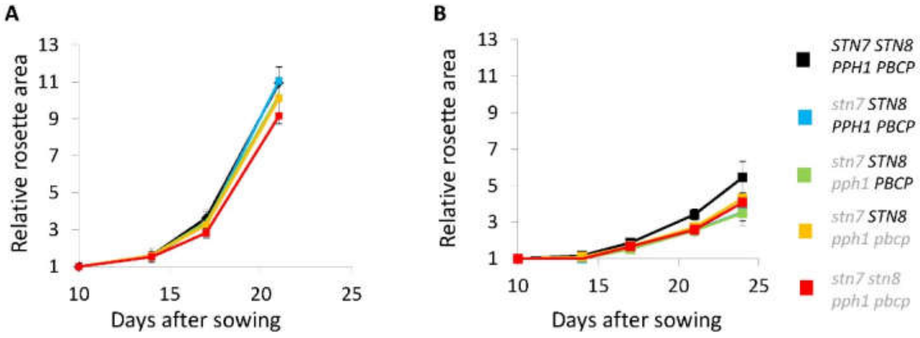

Supplementary Figure 7. Rosette area growth kinetics under constant and fluctuating light

Average rosette area relative to day 10 after sowing (10th day area = 1) for a set of 15 plants for each mutant line and the control wild type, grown under (A) constant light intensity (100 µmol photons m^{-2} sec^{-1}) and (B) fluctuating light with 4 minutes at 50 µmol photons m^{-2} sec^{-1} and 1 minute at 500 µmol photons m^{-2} sec^{-1}. Error bars represent the standard deviation of the mean.
